# Modulation of Immune-Inflammatory Responses in Abdominal Aortic Aneurysm: Emerging Molecular Targets

**DOI:** 10.1155/2018/7213760

**Published:** 2018-06-03

**Authors:** Hanrong Li, Shuling Bai, Qiang Ao, Xiaohong Wang, Xiaohong Tian, Xiang Li, Hao Tong, Weijian Hou, Jun Fan

**Affiliations:** ^1^Department of Tissue Engineering, School of Fundamental Science, China Medical University, Shenyang, Liaoning 110122, China; ^2^First Clinical College 99K, China Medical University, Shenyang, Liaoning 110122, China; ^3^Department of Cell Biology, Key Laboratory of Cell Biology, Ministry of Public Health, Key Laboratory of Medical Cell Biology, Ministry of Education, China Medical University, Shenyang, Liaoning 110122, China

## Abstract

Abdominal aortic aneurysm (AAA), a deadly vascular disease in human, is a chronic degenerative process of the abdominal aorta. In this process, inflammatory responses and immune system work efficiently by inflammatory cell attraction, proinflammatory factor secretion and subsequently MMP upregulation. Previous studies have demonstrated various inflammatory cell types in AAA of human and animals. The majority of cells, such as macrophages, CD4+ T cells, and B cells, play an important role in the diseased aortic wall through phenotypic modulation. Furthermore, immunoglobulins also greatly affect the functions and differentiation of immune cells in AAA. Recent evidence suggests that innate immune system, especially Toll-like receptors, chemokine receptors, and complements are involved in the progression of AAAs. We discussed the innate immune system, inflammatory cells, immunoglobulins, immune-mediated mechanisms, and key cytokines in the pathogenesis of AAA and particularly emphasis on a further trend and application of these interventions. This current understanding may offer new insights into the role of inflammation and immune response in AAA.

## 1. Introduction

Abdominal aortic aneurysm (AAA) is a common degenerative cardiovascular disease. This disease is generally caused by smoking, genetic diversity or variants, and atherosclerosis [1–3]. The majority of AAAs are detected in the infrarenal aorta, proximal to the aortic bifurcation [4]. AAA is a potentially lethal disease due to the risk of rupture [5]. Clinically, AAAs can be repaired using open surgical technique only when the diameter of aorta has surpassed 5.5 cm with a substantially increased risk of rupture [6]. Understanding the potential mechanism of AAA development and developing therapeutic strategies that modify the disease process of AAA is very important.

Vascular inflammation is the main initial factor of aortic aneurysm. In this process, a large number of exogenous immune cells, including lymphocytes, macrophages, mast cells, neutrophils, and natural killer cells, infiltrate into the tissue from adventitia to intima gradually, evoking a series of inflammatory response [7–11]. Infiltration of inflammatory cells and cellular elements produce and stimulate smooth muscle cells (SMC) to secret matrix metalloproteinases (MMPs), which are considered key enzymes directly related to AAA formation and progression [12, 13]. These enzymes destroy the stability and mechanical property of the aortic walls by modulating interstitial elastin and collagen [14–16], resulting in loss of smooth muscle cells in the aortic media and destruction of extracellular matrix (ECM) [17]. Inflammation is an important component of the immune system. The adaptive and innate immune systems have a great role in the initiation and propagation of the inflammatory response in aortic tissue. Recent increased knowledge suggests that immunological processes are involved in the pathogenesis of AAA [18–20]. In this view, we will discuss phenotypes of inflammatory cells, innate immune system, immunoglobulins, and key cytokines in the AAA disease and provide novel mechanistic insight for the development of immune-targeted therapies.

## 2. Innate Immunity

Innate immune system, also known as the nonspecific immune system, is the first line of defense against pathogenic invasion. In the pathological process of aortic aneurysm, a series of changes in the innate immune system including upregulation of TLRs (Toll-like receptors), activation of chemokine receptors, and deposition of complements were involved. We will show the most recent research progress in these areas and discuss particularly in the following paragraph.

### 2.1. TLRs in AAA

**Figure 1:**
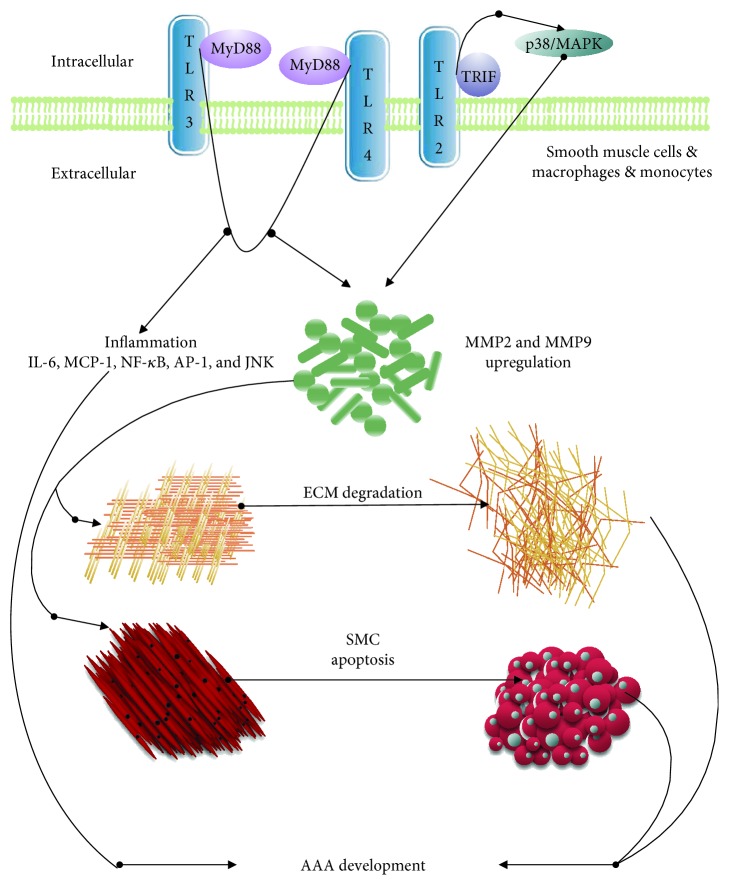
Possible mechanisms of TLRs in promotion of AAA development. The schematic diagram shows that TLR2 and TLR4 promote inflammation and MMP expression, and TLR3 promotes MMP expression in the aortic wall during aneurysm development.

TLRs play a fundamental role in several of inflammatory response and innate immunity process. As the initiating gate of innate immunity, pattern recognition receptor (PRR) activation is a start of all the subsequent immune responses [21, 22]. One of the transmembrane subtypes of PRRs, TLR, is a researching hotspot in recent years on the pathological mechanism of AAAs. TLRs are expressed on inflammatory cells (such as macrophages, monocytes, and B lymphocytes), endothelial cells, and SMCs, and all of these types of cells contribute to the inflammatory response of aortas [23]. In general, myeloid differentiation primary response gene-88 (MyD88) and TRIF as the intracellular signaling adaptors were involved in the proinflammatory process initiated by TLR activation. Most TLRs, including TLR2 and TLR4, signal through MyD88. But TLR3 signals through TRIF. Only TLR4 signals through both MyD88 and TRIF [24]. Till now, about 9 kinds of TLRs were discovered [25, 26] and some of these subtypes work actively in AAA (Figure 1).

#### 2.1.1. TLR2

TLR2 is mainly implicated in the initiation and maintenance of the inflammatory responses of autoimmune diseases. Upregulation of TLR2 contributes to immune reactivity and aggravates the inflammatory response [19]. TLR2 pathway displays a strong proinflammation action in aorta. TLR2 deficiency will decrease the concentrations of proinflammatory cytokines, whereas anti-inflammatory interleukin 10 (IL-10) was elevated [27, 28]. In atherosclerosis, TLR2 was involved in the process of inflammation and matrix degradation. Recently, activation of the TLR2 pathway has also been confirmed accelerating AAA formation [29], and a series of reactions coinciding with the crucial pattern of how the AAAs generate proinflammatory and MMP secretion followed. However, blocking TLR2 decreased the expression of endogenous ligands interacting with TLR2, and consecutively decreased chronic inflammation, activity of MMP2/9, and vascular remodeling of AAA [30]. Compared with their inhibitors of MMPs and anti-inflammatory agents, TLR2 blocking may provide a new therapeutic method in AAA treatment.

#### 2.1.2. TLR4

TLR4 is recognized as a vital traffic joint in AAA progression in recent years [31–35]. TLR4 can promote AAA formation directly by upregulating the expression of MMP-2 and MMP-9. In the indirect pathway, TLR4 induces the progression of AAA by elevating proinflammatory chemokine like IL-6 and MCP-1, proinflammatory cells like M1 macrophages, and the c-Jun NH2-terminal protein kinase (JNK) pathway [36–38]. JNK-signaling pathway can regulate inflammatory responses and is mainly activated by a series of phosphorylation [39]. TLR4 is an upstream of the JNK-promoting pathway [33, 38, 40]. The JNK pathway enhances MCP-1 expression and inflammatory cell infiltration [41]. Our investigation and other groups show that blockade of this pathway by its inhibitor, SP600125 or curcumin, can inhibit secretion of MCP-1, MCP-2, and MMP-9, thereby attenuating aortic aneurysm formation [42–44]. Shang et al. found MyD88-dependent TLR4 pathway participants in AAA progression. In this experiment, tanshinone IIA significantly decreased the overexpression of TLR-4, MyD88, phosphorylated nuclear factor *κ*B (pNF-*κ*B), and phosphorylated I*κ*B*α* (pI*κ*B*α*) in AAA induced by elastase perfusion [32]. Nevertheless, Owens et al. report that MyD88 slows down AAA formation independent of signal from TLR4 or TLR2. Given the critical roles of MyD88 and TLR4 in AAA, bone marrow transplantation is performed to determine whether the effect of MyD88 or TLR4 deficiency on AngII-induced AAA is mediated by cells of the hematopoietic lineage. MyD88 deficiency in bone marrow-derived cells profoundly reduces AngII-induced AAA. However, TLR4 deficiency in bone marrow-derived cells has no effect on AAA [45]. The difference appears probably due to the different inducer for the AAA model in use. In the future, a more detailed upstream and downstream of the TLR4 in AAA are needed to explore.

#### 2.1.3. TLR3

TLR2 and TLR4 have been shown to significantly induce atherosclerotic lesion and AAA by promoting macrophage recruitment and expression of inflammatory factors. However, TLR3 deficiency has no effect on aortic cytokine/chemokine expression [24, 46]. Ishibashi et al. discover that matrix-degrading action of TLR3 is partly mediated by modulation macrophage MMP-2 and -9 activities. The study highlighted that TLR3 signaling may increase MMP-2 activity by the p38/MAPK pathway [46]. However, collagen type I as an important structural component of plaque caps was not studied. In the future study, the role of TLR3 on collagen type I degradation should be investigated.

### 2.2. Chemokine Receptors

Chemokines are critical for the function of the innate immune, which own the ability to induce chemotaxis of immune cells after activation of the innate immune system [47]. According to the chemokine subclass, chemokine receptors, a large family of G protein-coupled receptors (GPCRs) [48], are classified into CR, CCR, CXCR, and CX_3_CR with a large variety of distribution and function in AAA [49]. Chemokines start a series of inflammatory reaction in AAAs. CXCR4 is believed to contribute to the AAA formation. When the receptor is blocked, the progression of AAA is attenuated [50, 51]. However, increased expression of CXCR4 in bone mesenchymal stem cells (BMSCs) might improve the treatment of AAA. Further studies are required to clear the detailed mechanism [52]. In TAA patients, a high concentration of CXCR3 ligand chemokines is detected in plasma. CXCR3 are the proinflammation chemokine receptor in AAA by attracting CD45-positive cell infiltration [53, 54]. Blockade of C-X-C motif ligand 1 (CXCL1) receptor, CXCR2, will attenuate tissue damage through inhibition of neutrophil recruitment [55, 56]. In aortas, CXCR2-neutralizing antibody obviously prevented the expansion and rupture of the dissected aorta by preventing neutrophil infiltration and reducing IL-6 expression [57]. As to another important chemokine receptor, CCR2, activation of CCR2 mediates the inflammation in AAA [58, 59] and this may be achieved by attracting mast cells to the tissue. One of its ligands, CCL2 (MCP-1), plays a crucial role in macrophage chemotaxis [60].

### 2.3. Complement

Complement factors are the major proinflammatory components of the innate immune system. Although as one of the nonclassical complement pathway, the complement alternative pathway contributes a lot to the AAA formation. C3 deposition is recognized as a cause of the subsequent reactions in AAA [61]. Zhou et al. discovered that IgG antibodies in plasma were able to activate the complement alternative pathway by inducing C3 deposition in AAA [62]. In their later study, they find that the IgG antibodies binding to fibrinogen can lead to AAA formation by activating the complement lectin pathway [63]. Another lectin pathway activator, ficolin-3, was also demonstrated to contribute to AAAs [64]. This may provide the evidence that the C3-inducing AAAs are not specific, and some proper anti-C3 drugs may work in attenuating the development of AAAs. Another complement component C4d, however, shows a protective role in some inflammatory aortic disease such as aortic dissection [65].

## 3. Immune Cell Infiltration in AAA

### 3.1. Macrophages

Macrophages play an important role in the innate and adaptive immune responses. Macrophage infiltrating into aortic tissue and secreting matrix degradable substance directly contributes to AAA formation [8, 66, 67]. Macrophages may recruit to the AAA area through an “outside-in” pattern which means infiltration initiates from the adventitia [68].

Macrophages have a great number of subtypes in which M1 phenotype and M2 phenotype play a major role in AAA. The M1/M2 ratio imbalance can promote the AAA development. M1 macrophages are proinflammatory, while M2 macrophages are anti-inflammatory [69–71]. M2 macrophages may achieve the anti-inflammation effect by release of IL-10 and profibrotic factors such as TGF-beta [72]. The protective effect of TGF-beta also involves a critical role in the control of excessive monocyte/macrophage activation, as monocyte depletion inhibits AAA formation [73]. M1 or M2 macrophage polarization plays an important effect in regulating chronic inflammatory process. The infiltrating M2 macrophage will convert to M1 macrophage and vice versa in certain circumstances [74]. CD4(+)CD25(+)Treg cells play a key role in the macrophage-to-M2 switching [75, 76]. So, intervention of preventing M2 to M1 transition or promoting macrophage switching into M2 may help a lot in AAA treatment.

#### 3.1.1. Cytokines Modulating Macrophage Infiltration

Granulocyte-macrophage colony-stimulating factor (GM-CSF) was once reported being able to change mesothelial cells into macrophage-like cells by an autocrine pattern [77]. GM-CSF gene expression was also associated with macrophage densities in the arterial wall [78]. Constantly increasing secretion of GM-CSF may trigger aortic aneurysm [79]. The study shows that GM-CSF is the key regulator of AAA. If the GM-CSF pathway is blocked, macrophage infiltration and MMP-9 secretion will decrease [80]. CD4+ T cells secrete more GM-CSF in smad3−/− mice compared with WT mice. Deficiency of smad3 in genes contributes to aortic aneurysm maybe through GM-CSF pathway [81].

MCP-1 (monocyte chemotactic protein-1), also called CCL-2, is a kind of c-c chemokine secreted mainly by inflammatory cells and endothelial cells [82]. It is positively correlated with macrophage infiltration into aortic walls and acts as a promoter of AAA formation and development [78, 83, 84]. Usually upregulation of MCP-1 occurs early than the chronic inflammatory responses [85]. Our previous study found that MCP-1 was involved in aortic aneurysm development. MCP-1 secreted by SMCs could promote AAA progression by enhancing MMP-9 production [86, 87]. The apoptotic SMCs attract monocytes and other leukocytes by producing MCP-1. However, MCP-1-primed macrophages will further elicit aortic SMC apoptosis [88, 89]. MCP-1-promoted AAA may also be achieved by enhancing macrophage infiltration and cytotoxicity as well as promoting SMC phenotype transformation and apoptosis [85, 90, 91]. IL-6 is a proinflammatory cytokine that can contribute to SMC apoptosis and modulate extracellular matrix by MMP enhancement [92, 93]. There is a regulatory loop between IL-6 and MCP-1. Recent study shows that IL-6 can promote macrophages secreting MCP-1 and, in turn, MCP-1 has a positive feedback on IL-6 through the p38 pathway [93]. In the AngII-induced mouse model, IL-6 and MCP-1 are upregulated. Lacking either IL-6 or MCP-1 receptor CCR2 will reduce the early onset of aortic dissections. The enhancement of MCP-1 and IL-6 can promote macrophage secreting CD14 and CD11b, which in turn can induce MCP-1 and MMP-9 expression [94]. At the same time, researchers identify that CD14 plays the crucial role in promoting the macrophage precursor recruitment in early AAA walls [67].

### 3.2. T Cells

#### 3.2.1. Th Cells

CD4+ Th cells include two main types, Th1 cells and Th2 cells. Th1 cells mainly secrete cytokines including IL-2 and INF-*γ*, while Th2-characteristic cytokines include IL-4 and IL-5 [95, 96]. Both types of cells regulate each other by these cytokines, and the Th1/Th2 ratio is dependent on the environment and inflammatory response [96]. Both Th1 cells and Th2 cells can contribute to vascular inflammation [97]. In most cases, Th1 cells play an anti-inflammatory role and Th2 cells play a proinflammatory role [98, 99]. A high ratio of Th2 cells in AAA was observed, and a dysfunctional IL-4 expression will reduce AAA formation [100, 101]. Th2 polarizing induced by CD19 treatment in mice alleviates the macrophage infiltration and vascular inflammation [102]. INF-*γ* secreted by Th1 cells may attenuate AAA formation and development [103]. Blockade of the INF-*γ* pathway will lead to sequential severe AAA formation with increased expression of MMP-2 and MMP-9 [100]. Cytokines of Th2 cells may promote collagenolytic and elastolytic activation while Th1-characteristic cytokine reduces MMP expression. On the contrary, Galle et al. find that Th1 cells are the prominent in fresh T cells isolated from AAA tissue with high expression of INF-*γ*, which suggests that INF-*γ* contributes to AAA formation [104]. Another study also demonstrates that INF-*γ* deletion attenuates MMP expression and inhibits aneurysm development. The attenuation function of INF-*γ* may come from other coacting signaling in Th1 cells [105]. The differences are potentially attributable to different animal models studied. The INF-*γ* effect on the development of AAA should be further explored in the same animal models, disease stage, and anatomical areas in the future study. The regulation of Th cell polarization will be an investigation direction in AAA treatment.

#### 3.2.2. Regulatory T (Treg) Cells

Treg cells as the T cell subpopulation are engaged in sustaining immunological self-tolerance and homeostasis, which are essential for preventing autoimmune diseases and limiting chronic inflammatory diseases [106]. The transcription factor Foxp3 helps Treg cells complete the specification to control immune responses. Foxp3(+) Tregs may prevent AAA formation by inhibiting local inflammation in the aortic wall. Genetic depletion of Foxp3(+) Tregs significantly increases the mortality of AAA [107]. It is always thought that functional Treg cells limit AAA development by secreting the inhibitory cytokines [108–110], such as IL-10, which plays key effect in reduction of cell death, inhibition of vascular smooth muscle cell proliferation, inhibition of macrophage function, and reduction in inflammatory cell recruitment. Cytokine IL-10 secreted by the Treg cells acts in the pathogenesis of AAA and suppresses inflammatory response [111–113]. TGF-beta, secreted by Treg cells, is confirmed that it can protect AAA progression [73]. Genetic variation of the TGF-beta pathway leads to AAA development and contributes to multiple syndromic presentations of aortic aneurysm [114, 115]. In contrast, the recent study also reports that deficiency of TGF-beta signal prevents AAA formation [116]. To understand the concreted mechanism, the studies should be to investigate the TGF-beta isoform involved in AAA formation and delete it in a cell-specific manner in mice.

Some cytokines can act back on Treg cells and regulate their function. A recent study finds that impaired secretion of TGF-beta results in number loss of Treg cells. Once monocytes and B cells have an impaired capacity in inducing Foxp3 upregulation of Treg cells, exogenous TGF-beta can rescue the function [117]. Flores-García et al. also find that Treg cells have an immunosuppressive activity on CD4+ T cell-dependent TGF-beta [118]. By releasing IL-10, IL-10-producing B cells are able to enhance Treg cell function and convert T effector cells into Treg cells [119]. Other cytokines such as IL-33, in collaboration with IgE, can also stimulate expansion of Treg cells [120, 121].

Recently, Balmert et al. have already succeeded in prohibiting allergic contact dermatitis by Treg induction. They use degradable microparticles containing TGF-beta, IL-2, and rapamycin to sustain a microenvironment to promote Treg cell differentiation [122]. The above evidence shows that the enhancing expansion and differentiation of Treg cells stimulated by cytokines may be a new therapeutic goal for AAA.

#### 3.2.3. CD8+ T Cells

CD8+ T cells are important in cell-mediated toxicity. Cytotoxic CD8+ T cells have been implicated in targeting vascular endothelial and smooth muscle cells [123]. Yet the study on their role in AAA is few. In one study, CD8+ T deficiency significantly promotes elastase-induced AAA formation [124]. Another report shows that modulation of the function CD8+ T cells through reducing macrophage infiltration and Th17 cell polarization can attenuate the AAA induced by AngII.

### 3.3. B Cells

B cells can be divided into two developmentally distinct groups, B1 and B2 cells. B1 cells play crucial roles in the process of innate immunity, while B2 cells are the conventional players in adaptive humoral immunity [125]. In AAA, IgM, IgG, and C3c deposits are detected in the fibrous zone, which indicates that pathogenic B cell response is involved in the pathogenesis of AAA [126]. B cells in AAA are mainly specifically recruited to the adventitia of the aortic wall after stimulation [127]. B2 cells are the largest constituent of B cells in mouse AAA [128]. In atherosclerosis, B1 cells are protective via production of natural antibodies IgM, whereas B2 cells are proatherogenic via activation/proliferation of T cells. The recent study found that B cell deficiency could increase Treg cell infiltration in AAA tissue and inhibit AAA formation. In their study after anti-CD20 treatment, both wild type and apolipoprotein in E-knockout mouse model appear significant B1 and B2 depletion. Sequentially, higher number of dendritic cells appeared in aortas. Treg cell number is increased, but proinflammation genes are downregulated [128]. Another study also supports this result, which demonstrates that angiotensin II mobilizes monocytes from spleen to aorta in a B cell-dependent manner and promotes AAA formation in the apolipoprotein E KO mice [129]. However, one group finds that B2 cells from spleen of 8- to 10-week-old wild-type mice could suppress experimental aortic aneurysm of muMT mice by upregulating Treg cells and decreasing the number of aortic-infiltrating mononuclear cells [130]. It is possible that B2 cell transplantation might produce the protective antibodies. The differences of above results are needed to further explore the paradox immune response in muMT and anti-CD20 antibody-mediated B cell depleted mice.

### 3.4. NK Cells

Natural killer cells have shown the role in the development of chronic inflammatory responses. Apart from macrophages, T cells, and B cells, NK cells were significantly increased in the peripheral blood in AAA patients, which resulted in the increasing of cytotoxic activity and contributing the AAA formation [10]. NK cells can produce the proinflammatory cytokines such as IL-2 and INF-*γ* [131, 132]. Evidence indicates that the NK pathway is activated in AAA. One study shows that TNF*α* level is increased in AAA patients, and T cells isolated from AAA patients produce more TNF*α* [133, 134]. Recently, the protein expression of the NK cytotoxic signaling pathway is identified. In AAA tissues, two important NK pathway proteins (HCST and GRZB) are found expressed in CD8+ T cell and macrophage that participating in this pathway [135]. However, the exact role of NK cells in AAAs is still unclear.

### 3.5. Mast Cells

Mast cells are implicated in a number of inflammatory diseases through releasing of inflammatory mediators, serglycin and other proteoglycans, and proteases [136]. In human and animal AAA, the mast cells have been identified [9]. Interventions of mast cells such as tryptase deficiency, chymase deficiency, and mast cell functional substance antagonists attenuated the formation of AAA [137, 138]. In a recent human AORTA trial, three doses of the mast cell inhibitor pemirolast are given to 326 patients and AAA growth is monitored over 12 months; the result demonstrates that AAA growth rates are similar in patients receiving placebo and different doses of pemirolast, which concludes that pemirolast cannot retard the growth of medium-sized AAAs [139]. The effect of mast cells in the AAA and the validity of mast cell inhibition used to develop effective medications for AAA need to be cleared.

### 3.6. Neutrophils

Neutrophils have already been recognized as one of the initial contributors in AAA formation [11] via secreting some particular ECM-degrading enzymes such as neutrophil collagenase (MMP-8) and neutrophil protease [140, 141]. In adventitia neutrophil recruitment and activation, neutrophil-derived IL-6 enhances the adventitial inflammation that leads to aortic rupture [57]. Recent studies have detected an elevated level of neutrophil gelatinase-associated lipocalin (NGAL), a protein expressed by polymorphonuclear neutrophil which is considered an activated form of neutrophil [142]. NGAL is also a potential indicator for evaluation in aortic aneurysm repair [143]. Further studies are needed to understand the relationship between the NGAL level and AAA presence and growth.

Neutrophil extracellular traps (NETs) are originally identified as an innate immune response to bacterial infection [144]. In human AAA, neutrophil activation is also associated with NET formation in the intraluminal thrombus (ILT) [145]. IL-1*β*-induced NET formation promotes the development of AAA [146]. Neutrophil protease-mediated NET release contributes to elastase-induced AAA through plasmacytoid dendritic cell activation and type I interferon production [141].

## 4. ILT in AAA

In about 75% of clinically relevant AAA patients, the aneurysm lumen wall is covered by ILT [147]. ILT is a complex fibrin network and contains inflammatory cells, chemokines, and proinflammatory cytokines as well as ECM constituents [148–151]. ILT has been shown to be related with aortic wall weakening and a higher level of immunoinflammation in the AAA [152]. The volume of ILT is associated strongly with AAA size and growth in patients [153]. Recent studies demonstrate that the proinflammatory cytokines, reactive oxygen species, and proteases in the thrombus play a significant role in the development of human AAA [150, 151, 154]. In aortic aneurysms induced by AngII in the ApoE−/− mouse, the thrombus within the aortic wall is often observed [155]. The blood-ILT interface releases biological mediators which will activate the platelets and the coagulation cascade [151]. Anticoagulants fondaparinux treatment can reduce intramural thrombus formation, inflammation, and growth of experimental aortic aneurysm in the mouse model [154].

## 5. Immunoglobulins

B cells (and/or dendritic cells) present that antigen to T cells, activated T cells, and B cells interacts to promote the activation, proliferation, and differentiation of B cells. After activation, B cells in the germinal centers experience class switching and affinity maturation to become plasma cells that secrete large amounts of highly specific antibodies.

### 5.1. IgG

IgG4 is the least abundant subclass among the IgG antibodies in the human body, but its level is associated with a series of vasculitis syndrome [156, 157]. One of the most common manifestations is inflammatory aortic aneurysm (IAA). Researchers find IgG4-positive plasma cell infiltration in the aortic wall of IgG4-related AAA [158–160]. The high level of serum IgG4 contributes to the aortic dilation [161]. IgG4-related AAA is one symptom of IgG4-related disease (IgG4-RD), a systemic inflammatory disease with a high level of serum IgG4, instead of an isolated disorder [162, 163]. Compared with the non-IgG4-related AAA, IAA shows a significant increase in the number of infiltrating IgG4-positive cells and the incidence of a disrupted follicular dendritic cell network in lymph follicles [164, 165], which indicates that IAA is not a simple inflammatory aorta. In general, patients with IgG4-related inflammatory aortic aneurysm have an allergic constitution [166]. Vasculitis caused by a high serum level of IgG4 is always treatable [158]. B cell depletion therapy acts well on IgG4-related AAA [128]. However, steroid therapy did not work well [167].

### 5.2. IgE

According to previous studies, high IgE concentration tends to promote coronary atherosclerosis and dilation [168–171]. Generally, it is always considered that IgE participates in artery inflammatory disease mainly through activating mast cells. However, a recent study shows that IgE participates the aortic aneurysm formation by acting on not only mast cells but also on CD4+ T cells and macrophages [9]. IgE induces CD4+ T cell production of IL-6 and IFN-*γ* but reduces the production of IL-10 [9]. This process may be similar to pulmonary inflammatory disease such as asthma [172–174].

### 5.3. IgM

In 2014, Villar et al. have found an intimate positive correlation between IgM and inflammatory disease [175]. Diepenhorst et al. also detected a promoting role of IgM antibodies in inflammation in acute myocardial infarction (AMI) patients [176]. In secreted IgM deficiency mouse model, atherosclerosis was facilitated by IgE [170]. However, natural IgM antibodies produced by B1 cells show a protective role in atherosclerosis and artery remodeling [177, 178]. IgM was also detected in the adventitia of AAA [126]. But its role in AAA formation and development is still unknown.

## 6. Immune Regulation Application

The outcome of targeting the inflammatory cells, innate immune system, and immunoglobulins in AAA has been reviewed (Table 1). Understanding and developing new strategies that regulate immunity will provide useful therapeutic targets for AAA.

**Figure 2:**
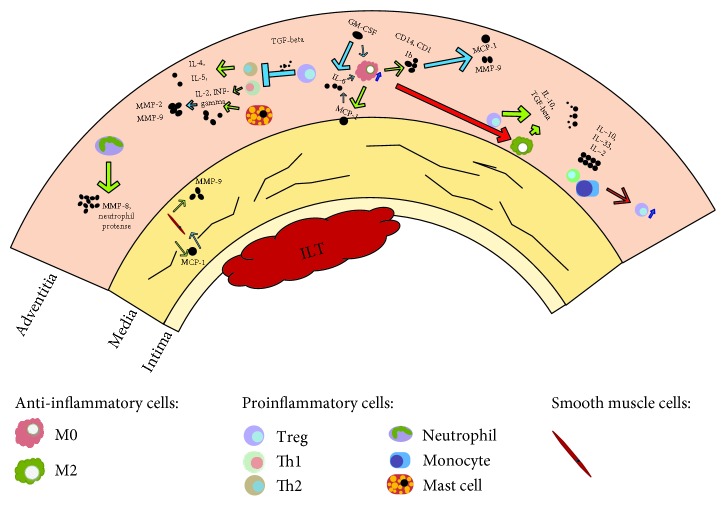
The roles of immune cells in the formation of AAA. The wall of AAA consists of plenty of inflammatory cell infiltration. Macrophages can be divided into two major phenotypes, M1 and M2 cells. M1 macrophages are proinflammatory. M2 macrophages are anti-inflammatory. CD4+ Th cells include two major types, Th1 and Th2 cells. Th1 cells mainly secrete cytokines including IL-2 and INF-*γ*, while Th2-characteristic cytokines includes IL-4 and IL-5. Neutrophils secrete collagenase (MMP-8) and neutrophil protease to degrade ECM. Mast cells produce cytokines, chemokines, and proteases, which further activate MMPs. The inflammatory cells release inflammatory mediators, which will result in breakdown of medial elastin and smooth muscle cell (SMC) apoptosis. Most aneurysms are covered by an intraluminal thrombus (ILT), and the presence of blood in the ILT is associated with AAA rupture.

Lots of studies have shown inflammatory cell infiltration in the AAA. The prominent of the cell types, such as macrophages and T cells, plays a significant role in the progression of AAA. Intervention of preventing M2 to M1 transition or promoting macrophage switching into M2 may help a lot in AAA treatment. About T cells, regulation of Th cell polarization (Th1/Th2 ratio) can be an investigation direction in AAA treatment. Another kind of T cell, Treg cells have the anti-inflammation ability. The investigations provide data which are beneficial to the treatment of AAA. Enhancing expansion and differentiation of Treg cells stimulated by cytokines may be a new therapeutic method for AAA (Figure 2).

In normal physiological conditions, B cells play crucial roles in innate immunity and humoral immunity. Under pathological environments, T cells are activated and then stimulate B cells to produce the diseased antibodies in response to stimulations. The diseased antibodies take effect in the inflammatory process [179]. Recent studies refer that the B cells were involved in the AAA. Removing B cells prevents the development of AAA. However, delivery of B2 cells from the young wild-type mice to the AAA mice increases the number of Treg cells and also inhibits the formation of AAA. It seems that the results are paradoxical. B cell function in AAA might be impaired. The immunoglobulins secreted by B cells are pathological and lost the normal function. It is possible that B2 cell transplantation might produce the healthy protective antibodies. In the future studies, the B cell number and immunoglobulins should be monitored and analyzed in the development of AAA. Although the related experiments on B cell effect in AAA are few, modulation of B cells might bring a new field for AAA treatment.

Immunoglobulins in blood own an extensive variety of recognizing ligands and functions. They can greatly affect the functions and differentiation of immune cells [180–182]. A high level of serum IgG4 contributes to the aortic dilation. B cell depletion therapy will be a good method to treat the IgG4-related AAA. Intravenously applied normal polyclonal immunoglobulins (IVIg) have great therapeutic applications in the treatment of autoimmune, infectious, and inflammatory diseases [183]. Immunoglobulins can hopefully be a new therapy target in these aortic inflammation diseases. Taking good advantage of the effect among immunoglobulins in the immunologic therapy can be another task.

Innate immune system such as Toll-like receptors (TLRs), chemokine receptors and complements are recently shown to regulate immunological processes leading to the formation and progression of AAAs as well as to other cardiovascular pathologies. Most recent work highlights the significance of TLRs in AAA development. TLR2 and TLR4 promote the inflammation and matrix degradation by upregulation of MMP expression in AAA. Blockage of TLRs may serve as a potential therapeutic strategy for AAA.

## 7. Conclusions

As the previous study demonstrates, inflammation plays a vital role in AAA formation, development, and progression. The immune system also participates in regulation control of the AAA pathological process and has a profound effect on the AAA-related inflammatory reactions. Therefore, it is very important to understand the immune-inflammatory responses in abdominal aortic aneurysm and search the potential molecular targets in AAA. Although a good deal of strategies has been proposed, the clinical practicability is still lack of testing. The validity requires further clinical validation.

## Figures and Tables

**Table 1:** Treatment effects of different immune therapeutic targets on the AAA progression.

Target	Treatment effect	Agent	Model	Reference
Immune system	Decrease aortic dilatation	Immunosuppressive agents	Elastase-induced rat aneurysm	[18]
TLR2	Decrease chronic inflammation, vascular remodeling and AAA formation	TLR2-neutralizing mAb	AngII-induced mouse aneurysm	[30]
TLR4	Repress aneurysm recurrence	Alginate oligosaccharide	Aneurysm patients	[31]
TLR4/MyD88	Attenuates AAA formation	Tanshinone IIA	Elastase-induced rat aneurysm	[32]
TLR4/JNK	Inhibit experimental AAA development	Rosiglitazone	AngII-induced mouse aneurysm	[33]
CXCR4	Suppress AAA formation and progression	AMD3100	CaCl_2_-induced mouse aneurysm	[50]
CCR2 monocytes	Decrease aortic dilatation	Everolimus	Angiotensin II- (A2-) infused apolipoprotein E-deficient mouse	[58]
Complement alternative pathway	Prevent aneurysm formation	Properdin-free AP C3 convertase	Elastase-induced mouse aneurysm	[62]
M1/M2 macrophages polarization	Inhibit AAA formation	D-series resolvins	Elastase-induced mouse aneurysm	[70]
Foxp3(+) Tregs	Decrease incidence (52%) and mortality (17%) of AAA	Interleukin-2 complex	Apolipoprotein E-deficient mice fed a high-cholesterol diet with angiotensin II	[110]
B cells	Prevent experimental AAA formation	Anti-CD20 antibody	Elastase perfusion or angiotensin II infusion apolipoprotein E-knockout mouse	[128]
Mast cells	No difference with the placebo group	Pemirolast	Medium-sized AAA patient	[139]
Neutrophils	Inhibit experimental AAA formation	Antineutrophil antibody	Elastase-induced mouse aneurysm	[11]
NETs	Attenuate AAA formation	Cl-amidine, an inhibitor NET formation	Elastase-induced mouse aneurysm	[146]

## Abbreviations

AAA: Abdominal aortic aneurysm
MMPs: Matrix metalloproteinases
ECM: Extracellular matrix
TLRs: Toll-like receptors
PRRs: Pattern recognition receptors
MCP-1: Monocyte chemotactic protein-1
IL-10: Interleukin 10
IL-6: Interleukin 6
JNK: c-Jun NH2-terminal protein kinase
GM-CSF: Granulocyte-macrophage colony-stimulating factor
IRF: Interferon-regulatory factor
STAT: Signal transducer and activator of transcription
MyD88: Myeloid differentiation primary response gene-88
SMC: Smooth muscle cell
NGAL: Neutrophil gelatinase-associated lipocalin
AMI: Acute myocardial infarction
TNF-*α*: Tumor necrosis factor *α*
NK: Natural killer
CXCL1: C-X-C motif ligand 1
GPCRs: G protein-coupled receptors
Treg: Regulatory T cell
TGF-beta: Transforming growth factor-*β*
IFN-*γ*: Interferon-*γ*
IVIg: Intravenously applied normal polyclonal immunoglobulins
NETs: Neutrophil extracellular traps
ILT: Intraluminal thrombus.
